# Under-Coupling Whispering Gallery Mode Resonator Applied to Resonant Micro-Optic Gyroscope

**DOI:** 10.3390/s17010100

**Published:** 2017-01-06

**Authors:** Kun Qian, Jun Tang, Hao Guo, Wenyao Liu, Jun Liu, Chenyang Xue, Yongqiu Zheng, Chengfei Zhang

**Affiliations:** Key Laboratory of Instrumentation Science & Dynamic Measurement of Ministry of Education, North University of China, Taiyuan 030051, China; tsienkun@163.com (K.Q.); tangjun@nuc.edu.cn (J.T.); guohaonuc@163.com (H.G.); liuwenyao@nuc.edu.cn (W.L.); xuechenyang@nuc.edu.cn (C.X.); zhengyongqiu86@163.com (Y.Z.); zcfdalianfe@126.com (C.Z.)

**Keywords:** waveguides, planar, resonators, Sagnac effect, gyroscopes

## Abstract

As an important sensing element, the whispering gallery mode resonator (WGMR) parameters seriously affect the resonant micro-optic gyroscope (RMOG) performance. This work proposes an under-coupling resonator to improve the resonator’s *Q* value and to optimize the coupling coefficient to maximize the RMOG’s sensitivity. GeO_2_-doped silica waveguide-type resonators with different coupling coefficients were simulated, designed, fabricated and tested. An under-coupling ring resonator with a quality factor of 10 million is reported. The RMOG system was built based on this resonator and the scale factor was tested on a uniaxial high-precision rotating platform. Experimental results show that this resonator could improve the RMOG sensitivity by five times.

## 1. Introduction

The resonant micro-optic gyroscope (RMOG) is a novel opto-electronic hybrid integrated sensor with great potential to realize miniaturized all-solid-state devices and monolithic device integration [1,2]. The RMOG has received widespread attention for application in fields such as inertial navigation and attitude stabilization [3,4]. In the RMOG, planar waveguide ring resonators are commonly used as sensing elements, and thus the manufacturing of high-quality resonators has become a major issue for RMOG fabrication.

As an important component of the RMOG, the resonator plays a major role in improving the overall gyroscope performance. Various resonator parameters are directly related to the gyroscope’s sensitivity, including the quality factor (*Q*) and the effective area. Given the continuing drive to miniaturize these gyroscopes, the effective area of the resonant cavity is increasingly limited. Therefore, the determination of ways to optimize the cavity parameters within this limited area to maximize gyroscope sensitivity is one of the aims of this study.

Recently, a number of important achievements in the development of planar ring resonators for RMOG applications have been reported [5,6,7,8,9,10,11]. Vannahme et al. fabricated a 6-cm-diameter ring resonator on a LiNbO_3_ substrate with a *Q* factor of 2.4 × 10^6^ and subsequently built a gyroscope system that can detect a minimum rotation rate $\Omega_{\min}$ = 10°/s [12]. Ciminelli et al. designed and fabricated an InP-based spiral resonator with a 6 × 10^5^ *Q* factor and a footprint of approximately 10 mm^2^ [13]; the resolution of the gyroscope that was fabricated based on this resonator was 150°/h. They believed that the gyroscope’s performance could be enhanced by up-scaling of the spiral resonator. They also reported a silica-on-silicon spiral resonator with a *Q* value of 1.5 × 10^6^ [14]. Feng et al. fabricated a silica waveguide ring resonator with a *Q* factor of up to 1.4 × 10^7^, and a short-term bias stability of 0.0055°/s and a long-term bias stability of 0.013°/s were also reported [15]. 

The use of different materials and processes to improve the *Q* factor of the resonator appears to be the main path to improved gyroscope performance. Daryl, T. et al. reported the best-performing waveguide ring resonator with a *Q* factor of up to $4.6\;\times \;10^{7}$ [16]. A high-quality Si_3_N_4_ waveguide was fabricated via low pressure chemical vapor deposition (LPCVD) and chemically mechanically polished, with a propagation loss of 0.05 db/m. Francesco, D. et al. designed and fabricated a large-sized (26 mm diameter) InGaAsP/InP resonator with a *Q* factor of $7\;\times \;10^{5}$ [17]. Different values of the gap between the straight waveguide and the ring were discussed to achieve high resonant depth in their work. 

A disk and toroidal resonator on a chip always possess an ultra-high quality factor [18,19]. These types of resonators require an external independent coupler to couple light into them. Precise control of the gap between the coupler and cavity is required, which makes it difficult to precisely control the coupling state of the cavity. However, the coupling state is an important factor to be considered for a RMOG. The effect of the resonator coupling state on the gyroscope performance has seldom been studied so far. The WGMR has three distinct coupling states [20]: under-coupling (where *t* < *a*), critical coupling (where *t* = *a*) and over-coupling (where *t* > *a*), where *a* is the round-trip factor and *t* is the transmission coefficient. In this work, the relationship between the resonator coupling state and the RMOG sensitivity has been modeled, simulated and analyzed. An under-coupling resonator with a resonant depth of *h* = 0.75 was determined to be optimal for RMOG applications.

To verify this study experimentally, we designed a group of waveguide-type resonators in different coupling states, and accurately controlled the coupling coefficients of these resonators using micro-nano-finishing technology. A RMOG system was then built to test these WGMRs. The experimental results show that the under-coupled resonator with *h* = 0.75 produced the highest scale factor for the RMOG.

## 2. Principle and Simulation

**Figure 1:**
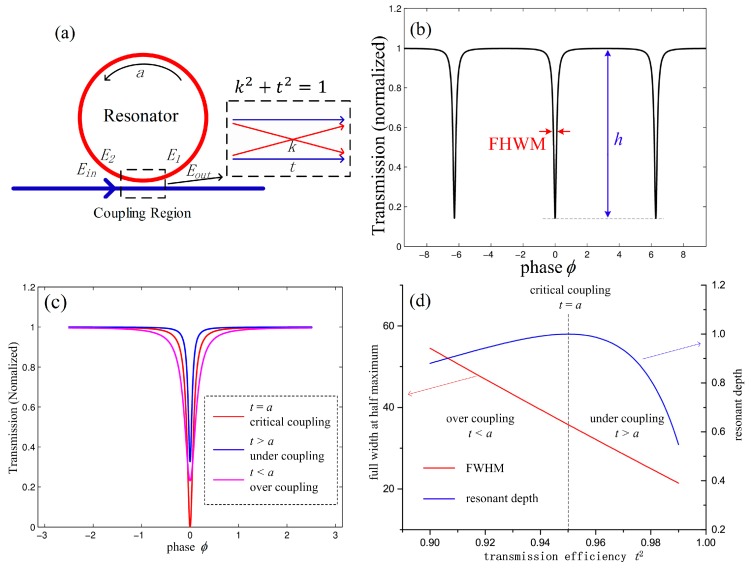
(**a**) Basic model of WGM resonator; (**b**) resonant spectrum of the resonator; (**c**) resonant spectra corresponding to critical coupling (*t* = *a*), under-coupling (*t* > *a*) and over-coupling (*t* < *a*); (**d**) full width at half maximum (FWHM) and resonant depth as functions of transmission efficiency *a*^2^ = 0.95.

With reference to Figure 1a, the WGM resonator coupling system can be described using the resonator round-trip factor (*a*) and the transmission coefficient (*t*) [21]. *E_in_* and *E_out_* are the input and output of the light fields, respectively; light is coupled into and out of the ring resonator via the coupling region. The field in the cavity *E*_1_ becomes *E*_2_ around the ring. According to the matrix analysis method of the ring resonator and the optical waveguide [22], the relationship of the fields *E_in_*, *E_out_*, *E*_1_, *E*_2_ could be expressed by:

(1) $$\left(\begin{array}{l} E_{out}\\ E_{in} \end{array}\right)=\left(\begin{array}{cc} t & -ik\\ -ik & t \end{array}\right)\left(\begin{array}{l} E_{2}\\ E_{1} \end{array}\right)$$

(2) $$E_{2}=ae^{i\phi }E_{1}$$

*k* and *t* are the coupling coefficient and transmission coefficient of the coupling region, respectively, ϕ=βL is the round-trip phase of the resonator, *L* is the perimeter of the resonator, and β is the propagation constant. Combining Equations (1) and (2), the power transmission *T* can be derived,

(3) $$T={\left|\frac{E_{out}}{E_{in}}\right|}^{2}=\frac{t^{2}+a^{2}-2ta\cos\;\phi }{1+t^{2}a^{2}-2ta\cos\;\phi },$$

Using Equation (3), the resonance curve can be drawn as shown in Figure 1b. The spectrum of the ring resonator is a downward absorption peak; the full width at half maximum ∆*f* and the resonant depth *h* of the absorption peak can be expressed as follows:

(4) $$\Delta f=\frac{c}{n\pi L}\arccos\;\frac{2ta}{1+t^{2}a^{2}},$$

(5) $$h=1-{\left(\frac{t-a}{1-ta}\right)}^{2},$$

where *c* is the speed of light in vacuum, and *n* is the refractive index of the resonator. Equation (3) shows that the coupling state of the resonator is determined by *t* and *a* (see Figure 1c) as follows: (i) for under-coupling (where *t* < *a*), the gap between the straight waveguide and the resonator is relatively large, and the light is not fully coupled into the resonator through the coupling region; (ii) for critical coupling (where *t* = *a*), the efficiency of the light coupling into the resonator is equal to the intrinsic attenuation of the resonator, and it can be seen that *h* = 1 at the critical point; (iii) for over-coupling (where *t* > *a*), the straight waveguide is closer to the resonator, and the larger coupling coefficient leads to increased light power losses. In waveguide-type resonators, the round-trip factor (*a*) is related to the propagation loss of the waveguide, and a lower transmission loss means a narrower resonance spectral linewidth and higher sensitivity for WGM resonator–based sensors; the transmission coefficient (*t*) can be adjusted by varying the gap between the straight waveguide and the resonator to produce different resonator coupling states and to obtain a high *Q* factor and a high resonant depth simultaneously. Figure 1d shows the relationship between the full width at half maximum (FWHM), the resonant depth and the transmission efficiency (*t*^2^), where the FWHM decreases with the increasing *t*^2^, and the resonant depth increases to a maximum value at the critical coupling point before decreasing.

Equations (4) and (5) illustrate the relationship between the full width at half maximum (Δf), the resonant depth (*h*) and the transmission coefficient (*t*), and the influence of the gap on the transmission coefficient (*t*) has been simulated using the finite-difference beam propagation method. Combining Equations (4) and (5), we can estimate the FWHM and resonant depth for the designed waveguide-type resonator.

A high resonant depth (*h*) means a high signal-to-noise ratio for the RMOG; the waveguide-type resonator has a smaller *Q* factor and a lower resonant depth in the over-coupling state, so the resonator should not be designed to operate in the over-coupling state. The under-coupling resonator has a higher *Q* factor but a lower resonant depth when compared to that at the critical coupling point. In our RMOG system, the laser light is modulated using triangular phase modulation, which is equivalent to square wave frequency modulation. The laser center frequency *f_0_* is modulated to a frequency of *f* ± ∆*f*. The RMOG output can then be expressed as [23]:

(6) $$I_{out}=I_{in}\left(\frac{r^{2}+a^{2}-2ra\cos\;\frac{2\pi \left(f+2\Delta f\right)}{FSR}}{1+r^{2}a^{2}-2ra\cos\;\frac{2\pi \left(f+2\Delta f\right)}{FSR}}-\frac{r^{2}+a^{2}-2ra\cos\;\frac{2\pi \left(f-2\Delta f\right)}{FSR}}{1+r^{2}a^{2}-2ra\cos\;\frac{2\pi \left(f-2\Delta f\right)}{FSR}}\right),$$

**Figure 2:**
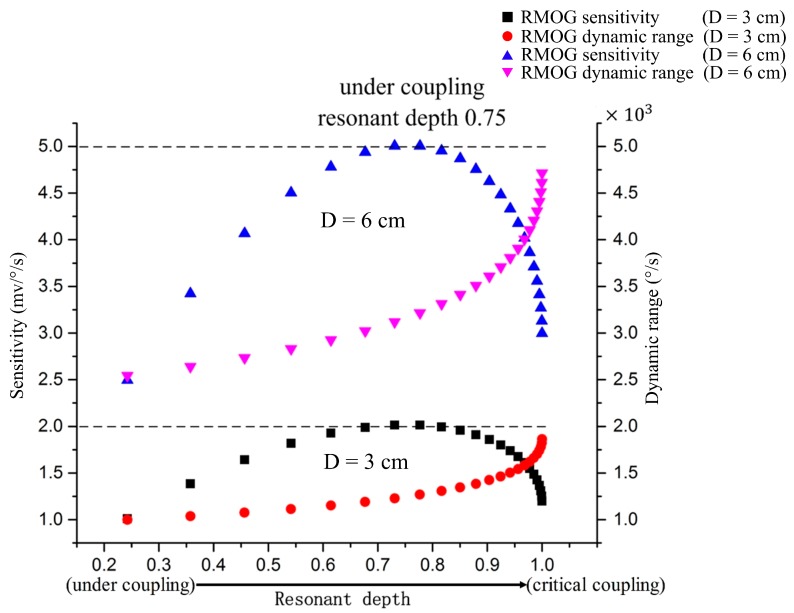
Gyroscope sensitivity and dynamic range as functions of resonant depth (as the coupling state varies from under-coupling to critical coupling).

When the coupling state varies from the under-coupling state (*t* → 0) to the critical coupling state (*t* = *a*), the resonant depth increases from 0 to 1. Figure 2 shows that the slope $\frac{dI_{out}}{d\Delta f}{|}_{\Delta f=0}$ of the demodulation curve has a maximum value at the point where *h* = 0.75, which means that the sensitivity of the RMOG has a maximum value when the waveguide-type resonator is in the under-coupling state and *h* = 0.75. Therefore, according to Equation (5), the design of the waveguide-type resonator for application to the RMOG should meet the following condition:

(7) $$t=\frac{2a+1}{a+2},$$

## 3. Experiment

**Figure 3:**
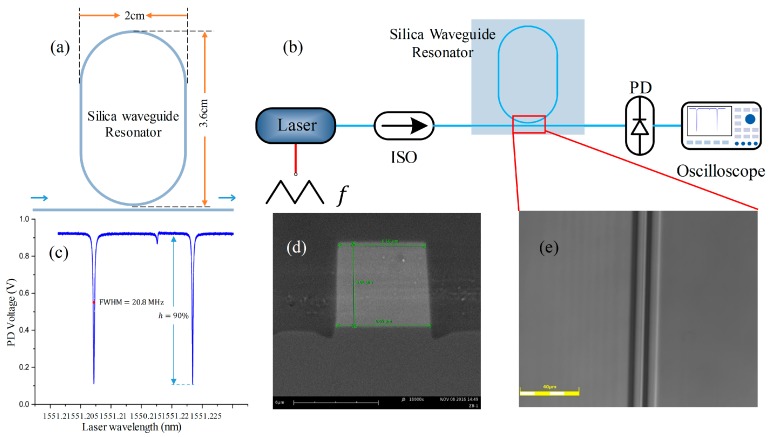
(**a**) Designed silica waveguide resonator; (**b**) test system used to measure the *Q* actor and the resonant depth; (**c**) test results for resonator with a 4.8 μm gap; (**d**) scanning electron microscope (SEM) image of waveguide cross-section; (**e**) coupling region viewed under confocal microscope.

We designed a group of silica waveguide-type resonators with gaps ranging in size from 3.8 μm to 5.2 μm (see Figure 3a). The refractive indices of the core and the overlay were *n*_1_ = 1.456 and *n*_2_ = 1.445, respectively. The waveguide core size was set at 6 × 6 μm^2^ to support single-mode transmission (the first-order mode was filtered by the bends in the waveguide). The relationship between the gap and the transmission coefficient (*t*) was also simulated using the beam propagation method (BPM), with results as shown in Table 1.

The resonators ware fabricated on a silicon substrate. First, a 10 μm-thick silica film layer was thermally grown on the silicon wafer as the lower cladding; this thickness of cladding was used to reduce the substrate leakage loss of the fundamental transverse electric (TE) mode. A six-micron waveguide core layer doped with GeO_2_ was then deposited on the lower cladding by plasma-enhanced chemical vapor deposition (PECVD). Doping GeO_2_ was done to increase the refractive index of the silica waveguide core film to 1.456. The waveguide-type resonator was fabricated by lithography and the reactive ion etching process before finally being covered with an upper cladding by PECVD. The material of the upper cladding was boron phosphate silicate glass, and the thickness was 10 microns. The melting point of the upper cladding layer was lower than the core and the lower cladding, and had better high-temperature fluidity, which ensured the steps were covered well. The doping concentration of boron and phosphate was adjusted to make the refractive index of the upper cladding equal to that of the lower cladding. The transmission loss of the silica waveguide was measured as α = 0.017 dB/cm, and thus the round-trip factor can be expressed as *a* = $\sqrt{10^{-\alpha L/10}}$, where α includes all loss contributions contained within the propagation loss of the waveguide and the radiation losses caused by the bends; in this case, the resonator length was *L* = 9.5 cm.

An experimental system (Figure 3b) was set up to measure the resonance spectrum of the WGMR. A tunable laser with an operating wavelength of 1550 nm and a spectral linewidth of 300 kHz was used to excite the WGMs, and a triangular voltage signal was applied to the laser for linear tuning of the laser frequency. The downward absorption peak could be observed on the oscilloscope (see Figure 3c).

**Figure 4:**
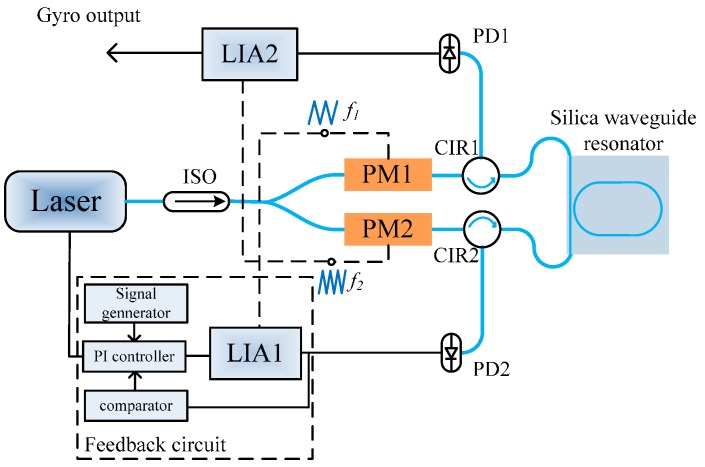
Schematic diagram of the RMOG.

A waveguide-type resonator-based RMOG system was set up based on the schematic shown in Figure 4. The laser has a central wavelength of 1550 nm, a narrow linewidth (100 Hz), and a sweep coefficient of 15 MHz/V. Two LiNbO_3_ phase modulators were used to modulate the clockwise (CW) and counterclockwise (CCW) optical signals. Two different modulation frequencies, *f*_1_ = 300 kHz and *f*_2_ = 555 kHz, were applied to the phase modulators to suppress any backscattered noise. Two circulators were used to couple the light into and out from the silica waveguide resonator. Photodiodes converted the light intensity signals to current signals, and then were converted to voltage signals by transimpedance amplifiers.

A feedback circuit was used to track the resonance frequency of the waveguide resonator. The demodulated signal from the lock-in amplifier (LIA1) was used to supply feedback to the frequency locking module to lock the laser’s central frequency to the resonance point of the resonator through a PI controller, and the other demodulated signal from LIA2 was used as the gyro output. With a triangular wave signal sweeping the frequency of the laser, the location of the resonant valley was determined. When the frequency tracking was active, the PI controller started to control the laser frequency to track the resonant frequency of the resonator. After the frequency tracking, the output of PD2 was always at the bottom of the resonant valley.

The prototype gyroscope was fixed on a uniaxial high-precision rotating platform. The resonator was placed in a thermostatic case, in which the temperature error is less than 0.001 °C. The scale factor of the RMOG was tested and fitted based on four different input angular velocities: ±40°/s, ±60°/s, ±80°/s and ±100°/s.

## 4. Result and Discussion

**Figure 5:**
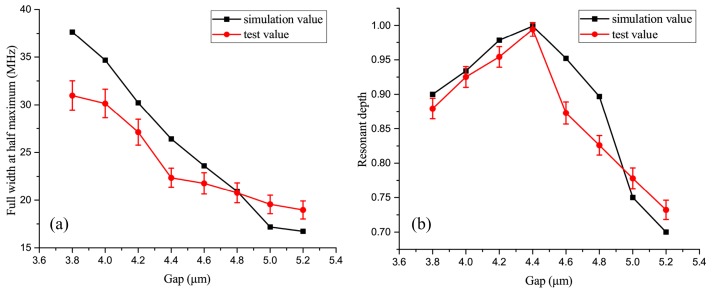
(**a**) Comparison of measured and simulated FWHM values for different gaps; (**b**) Comparison of measured and simulated resonant depth values for different gaps.

Figure 5a shows that the full width at half maximum (FWHM) of the resonator decreases with the increasing gap size, while the *Q* factor of the resonator increases as the coupling changes from over-coupling to under-coupling. The comparison shows that the *Q* factor of the 5.2-μm-gap resonator is double that of the 3.8-μm-gap resonator. Figure 5b shows that the resonant depth decreases after an initial increase, and that it reaches a maximum value at the critical coupling state. 

**Figure 6:**
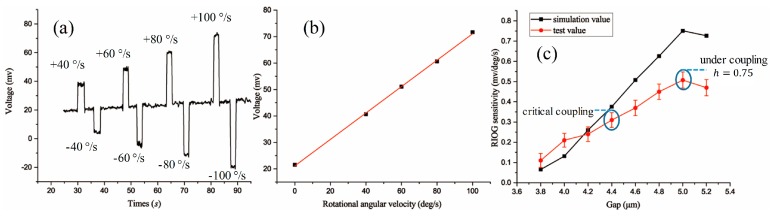
(**a**) Gyroscope signal with different rotational angular velocities; (**b**) fitting analysis of stair effects; (**c**) comparison of measured and simulated values of RMOG sensitivity for the different gaps.

We carried the rotation experiment under different rotational speeds with each resonator. The gyro output was observed as a voltage signal through an oscilloscope. The gyro step output waveforms caused by different rotational angular velocities are shown in Figure 6a. Scale factors that correspond to the resonators with the different gaps are shown in Figure 6c. The sensitivity of the RMOG increased almost linearly with the gap from over-coupling to critical coupling; this is because of the increasing *Q* factor and resonant depth. From critical coupling to under-coupling, *h* = 0.75, and the resonant depth started to reduce, but the sensitivity still increased due to the increase of the *Q* factor being the dominant factor. The final fitting results show that the RMOG sensitivity had a maximum value when the waveguide-type resonator was in the under-coupling state and *h* = 0.75. Compared with 3.8-μm-gap resonator, the RMOG sensitivity was improved by five times. If the gap of the resonator was further increased, higher *Q* values could be obtained, but the RMOG sensitivity could not be increased due to the sharp decline of the resonant depth. 

The comparison of Figure 5a,b with Figure 6c shows that the simulated and measured results display similar trends and agree well. These results prove that the under-coupling state can enhance the *Q* factor of the WGMR and improve the overall gyroscope performance. We have also determined the optimum coupling condition (Equation (7)) of the WGMR for the RMOG. This could be helpful in the application of WGMRs to RMOGs or other fields. 

However, the differences between the simulated values and the test values are mainly caused by waveguide propagation loss errors, gap errors caused by the photolithography process, and resonance spectrum measurement errors caused by the laser linewidth. Many additional factors, including backscattering noise, polarization fluctuation noise and Kerr effect noise, were also not suppressed well. These factors will be the focus of our future work.

## 5. Conclusions

In conclusion, an under-coupling resonator was proposed for use in resonant micro-optic gyroscopes. The relationship between the resonator’s coupling state and the gyroscope’s sensitivity was revealed. Both the simulated and experimental results show that an under-coupling resonator with a resonant depth of *h* = 0.75 would produce the highest sensitivity for the RMOG, and this has guided us to determine that a resonator design for RMOG applications should meet the following condition: $t=\frac{2a+1}{a+2}$. We designed and fabricated silica waveguide-type resonators with various gaps. A scale factor of 0.507 mV/°/s was achieved using an open-loop system, which is the highest value attained among all the resonators that we have designed, and this result is consistent with those of the simulations.

## Figures and Tables

**Table 1:** Transmission coefficient, full width at half maximum and resonant depth obtained by the simulation.

Gap (μm)	Transmission Coefficient	FWHM (MHz)	Resonant Depth (%)
3.8	0.9649	37.64	90
4.0	0.9690	34.68	93.39
4.2	0.9752	30.21	97.84
4.4	0.9807	26.42	99.9
4.6	0.9846	23.6	95.21
4.8	0.9884	20.91	89.69
5.0	0.9938	17.18	75
5.2	0.9945	16.72	70

## References

[1] Ciminelli C., Dell’Olio F., Campanella C., Armenise M.N. (2012). Photonic technologies for angular velocity sensing. Adv. Opt. Photonics.

[2] Dell’Olio F., Indiveri F., Innone F., Russo P.D., Ciminelli C., Armenise M. (2014). System test of an optoelectronic gyroscope based on a high *Q*-factor InP ring resonator. Opt. Eng..

[3] Barbour N., Schmidt G. (2002). Inertial sensor technology trends. IEEE Sens. J..

[4] Lefèvre H.C. (2014). The fiber-optic gyroscope, a century after Sagnac’s experiment: The ultimate rotation-sensing technology?. C. R. Phys..

[5] An P., Zheng Y., Yan S., Xue C., Wang W., Liu J. (2015). High-*Q* microsphere resonators for angular velocity sensing in gyroscopes. Appl. Phys. Lett..

[6] Mao H., Ma H., Jin Z. (2011). Polarization maintaining silica waveguide resonator optic gyro using double phase modulation technique. Opt. Express.

[7] Wang J., Feng L., Wang Q. (2016). Reduction of angle random walk by in-phase triangular phase modulation technique for resonator integrated optic gyro. Opt. Express.

[8] Feng L., Wang J., Zhi Y., Tang Y., Wang Q., Li H., Wang W. (2014). Transmissive resonator optic gyro based on silica waveguide ring resonator. Opt. Express.

[9] Jin Z., Zhang G., Mao H., Ma H. (2012). Resonator micro optic gyro with double phase modulation technique using an FPGA-based digital processor. Opt. Commun..

[10] Dell’Olio F., Conteduca D., Ciminelli C., Armenise M.N. (2015). New ultrasensitive resonant photonic platform for label-free biosensing. Opt. Express.

[11] Ma H., Zhang J., Wang L., Lu Y., Ying D., Jin Z. (2015). Resonant micro-optic gyro using a short and high-finesse fiber ring resonator. Opt. Lett..

[12] Vannahme C., Suche H., Reza S. Integrated optical Ti:LiNbO_3_ ring resonator for rotation rate sensing. Proceedings of the 13th European Conference on Integrated Optics (ECIO).

[13] Ciminell C., D’Agostino D., Carnicella G., Dell’Olio F., Conteduca D., Ambrosius H.P.M.M., Smit M.K., Armenise M.N. (2015). A High-*Q* InP Resonant Angular Velocity Sensor for a Monolithically Integrated Optical Gyroscope. IEEE Photonics J..

[14] Ciminelli C., Dell’Olio F., Campanella C.E., Armenise M.N. Numerical and Experimental Investigation of an Optical High-*Q* Spiral Resonator Gyroscope. Proceedings of the 2012 14th International Conference on Transparent Optical Networks (ICTON).

[15] Wang J., Feng L., Wang Q., Jiao H., Wang X. (2016). Suppression of backreflection error in resonator integrated optic gyro by the phase difference traversal method. Opt. Lett..

[16] Spencer D.T., Bauters J.F., Heck M.J.R., Bowers J.E. (2014). Integrated waveguide coupled Si_3_N_4_ resonators in the ultrahigh-*Q* regime. Optica.

[17] Dell’Olio F., Ciminelli C., Armenise M.N., Soares F.M., Rehbein W. Design, fabrication, and preliminary test results of a new InGaAsP/InP high-*Q* ring resonator for gyro applications. Proceedings of the 2012 International Conference on Indium Phosphide and Related Materials (IPRM).

[18] Armani D.K., Kippenberg T.J., Spillane S.M., Vahala K.J. (2003). Ultra-high-*Q* toroid microcavity on a chip. Nature.

[19] Lee H., Chen T., Li J., Yang K.Y., Jeon S., Painter O., Vahala K.J. (2012). Chemically etched ultrahigh-*Q* wedge-resonator on a silicon chip. Nat. Photonics.

[20] Matsko A., Ilchenko V. (2006). Optical Resonators with Whispering-Gallery Modes—Part I: Basics. IEEE J. Sel. Top. Quant..

[21] Cai M., Painter O., Vahala K. (2000). Observation of critical coupling in a fiber taper to a silica-microsphere whispering-gallery mode system. Phys. Rev. Lett..

[22] Poon J.K.S., Scheuer J., Mookherjea S., Paloczi G.T., Huang Y., Yariv A. (2004). Matrix analysis of microring coupled-resonator optical waveguides. Opt. Express.

[23] Ying D., Wang Z., Mao J., Jin Z. (2016). An open-loop RFOG based on harmonic division technique to suppress LD’s intensity modulation noise. Opt. Commun..

